# The Estimation of Critical Angle in Climbing as a Measure of Maximal Metabolic Steady State

**DOI:** 10.3389/fphys.2021.792376

**Published:** 2022-01-05

**Authors:** Jiří Baláš, Jan Gajdošík, David Giles, Simon Fryer

**Affiliations:** ^1^Faculty of Physical Education and Sport, Charles University, Prague, Czechia; ^2^Lattice Training Ltd., Chesterfield, United Kingdom; ^3^School of Sport and Exercise, University of Gloucestershire, Cheltenham, United Kingdom

**Keywords:** sport climbing, muscle oxygenation, near infrared spectroscopy, critical power, oxygen kinetics, finger flexors

## Abstract

**Purpose:** Sport climbing is a technical, self-paced sport, and the workload is highly variable and mainly localized to the forearm flexors. It has not proved effective to control intensity using measures typical of other sports, such as gas exchange thresholds, heart rate, or blood lactate. Therefore, the purposes of the study were to (1) determine the possibility of applying the mathematical model of critical power to the estimation of a critical angle (CA) as a measure of maximal metabolic steady state in climbing and (2) to compare this intensity with the muscle oxygenation breakpoint (MOB) determined during an exhaustive climbing task.

**Materials and Methods:** Twenty-seven sport climbers undertook three to five exhaustive ascents on a motorized treadwall at differing angles to estimate CA, and one exhaustive climbing test with a progressive increase in angle to determine MOB, assessed using near-infrared spectroscopy (NIRS).

**Results:** Model fit for estimated CA was very high (*R*^2^ = 0.99; SEE = 1.1°). The mean peak angle during incremental test was −17 ± 5°, and CA from exhaustive trials was found at −2.5 ± 3.8°. Nine climbers performing the ascent 2° under CA were able to sustain the task for 20 min with perceived exertion at 12.1 ± 1.9 (RPE). However, climbing 2° above CA led to task failure after 15.9 ± 3.0 min with RPE = 16.4 ± 1.9. When MOB was plotted against estimated CA, good agreement was stated (ICC = 0.80, SEM = 1.5°).

**Conclusion:** Climbers, coaches, and researchers may use a predefined route with three to five different wall angles to estimate CA as an analog of critical power to determine a maximal metabolic steady state in climbing. Moreover, a climbing test with progressive increases in wall angle using MOB also appears to provide a valid estimate of CA.

## Introduction

Sport climbing is a technical, self-paced sport, and the workload is highly variable and mainly localized to the forearm flexors. Both maximal finger flexor strength and endurance have been found to be strong predictors of climbing ability (Fryer et al., 2018; Michailov et al., 2018), with lead climbers demonstrating greater endurance and boulderers maximal strength and power (Fanchini et al., 2013; Fryer et al., 2017). The recent debut of competition format climbing at the Tokyo Olympics 2021 (the combined performance of speed, lead, bouldering) has highlighted the divergent requirements of different disciplines, forcing athletes to pay special attention to concurrent training of strength or power and endurance to improve their combined performance.

An ascent of a climbing route is rarely “standardised” with numerous changes in wall angle and speed, and also the types, shapes, orientation, and distributions of handholds, and opportunities for partial recovery during an ascent. As such, performance requires the interaction of multiple technical, tactical, neuromuscular, and metabolic factors (Orth et al., 2016; Saul et al., 2019). However, during training, climbers still seek to stimulate these factors in an isolated manner using intensity-controlled devices such as hangboards, campus boards, and climbing walls of different angles (Medernach et al., 2015; Levernier and Laffaye, 2019; Stien et al., 2021). Diagnostic and training methods for climbing-specific strength have been well described in the literature (López-Rivera and González-Badillo, 2012; Medernach et al., 2015; Michailov et al., 2018; Levernier and Laffaye, 2019; Lopez-Rivera and Gonzalez-Badillo, 2019; Philippe et al., 2019; Stien et al., 2021). In contrast, research on adaptations from endurance training is scarce (Lopez-Rivera and Gonzalez-Badillo, 2019). Endurance training in climbing requires systemic and localized adaptations (Thompson et al., 2014; Fryer et al., 2018), and ensuring appropriate intensity of exercise, particularly for the finger flexors, is challenging. Indeed, it has been shown that intensity control during climbing using measures typical from other sports, such as gas exchange thresholds, heart rate, and blood lactate, are not effective (Schöffl et al., 2006; Limonta et al., 2018; Baláš et al., 2021).

Only two studies have proposed a test to determine functional aerobic metabolic capacity in climbers using intermittent isometric handgrip contractions at differing intensities (Giles et al., 2019, 2020). The authors calculated critical force (CF), the force analog of critical power (CP) to determine maximal metabolic steady state for climbing-specific handgrip exercise (Poole et al., 2016; Jones et al., 2019). The CF tests proposed by Giles et al. (2019, 2020) are useful; however, they may only be applied to isolated forearm models and so far have only been tested for one specific hold size and work-recovery ratio, and therefore, their practical use is currently limited.

Applying the CP concept (Poole et al., 2016), its mathematical models to a whole-body climbing test may offer a potential solution to determine maximal metabolic steady state in climbing. Although climbing intensity has often been increased by elevating the velocity of an ascent (Booth et al., 1999; España-Romero et al., 2009; Rosponi et al., 2012), it has recently been shown that local muscle oxygen utilization may not be altered during faster climbing; however, it does rise with steeper wall angles (Gajdošík et al., 2021). Small incremental changes in climbing angle offer a valid means of altering the intensity of a climb while maintaining its multifaceted characteristics (Noé et al., 2001; Baláš et al., 2014). Combined with the measures of climbing time to exhaustion (TTE), it may be possible to calculate a “critical angle” (CA) analogous to CP (Poole et al., 2016). The CA should correspond to a metabolic transitional zone below which climbing does not induce task failure for a prolonged period, and above which fatigue occurs in a finite predictable period. Moreover, with an increased angle, more pronounced finger flexor contractions stimulate mitochondrial respiration and higher intramuscular pressure restricts capillary blood flow and, thus, muscle oxygen delivery (Fryer et al., 2013; Gajdošík et al., 2021). Recently, muscle oxygenation breakpoints (MOBs) have been measured locally using near infrared spectroscopy (NIRS) during an incremental climbing task (Baláš et al., 2021). These MOBs were suggested to represent an intensity around localized CP; however, they have not been associated with any systemic metabolic threshold indicators, and as such validation of such a MOB is needed.

Knowledge of CA in climbers may help coaches and researchers to set climbing intensities on routes with preset hold configurations (specific type, shape, orientation, and distribution of handholds and footholds) in the heavy or severe exercise domains during training; something, which would be extremely advantageous for training, yet is currently not possible. Moreover, the use of NIRS may allow for the instantaneous control of intensity during an ascent. We hypothesize, that if a climbing CA exists, the difference in intensity will also elicit changes in muscle oxygen dynamics. Moreover, climbing slightly over CA will lead to a finite and predictable time to failure, and climbing under the CA will not induce exhaustion for a prolonged, indefinite period. Consequently, the purposes of the study were to (1) determine the possibility of applying the mathematical model of CP to the estimation of a CA as a measure of maximal metabolic steady state in climbing and (2) to compare this intensity with the MOB determined during an exhaustive climbing task.

## Materials and Methods

### Participants

Twenty-seven sport climbers of an intermediate to advanced level [11–25 International Rock-Climbing Association (IRCRA) scale; 6a–8b French/Sport scale] volunteered (19 men: age 30.3 ± 8.5 years, body mass 70.5 ± 7.1 kg, height 177 ± 6 cm; 8 women: age 26.2 ± 3.0 years, body mass 57.4 ± 6.9 kg, height 169 ± 5 cm). Training characteristics of the participants reported during the initial questionnaire are depicted in Table 1. All participants were informed of the experimental risks and provided informed consent prior to the commencement of data collection. Climbers were healthy non-smokers who were not taking any vascular acting medication. The study conformed to the recommendations of World Medical Association and the Declaration of Helsinki and was approved by the Ethics Committee of Charles University, Faculty of Physical Education and Sport under the N*^o^* EK 61/2019.

**TABLE 1:** Performance and training characteristics (mean ± SD) in male and female climbers.

	Males	Females	Differences	
	*N* = 19	*N* = 8	*P*	Cohen’s *d*
Climbing ability lead (IRCRA scale)	17.9 ± 4.2	16.3 ± 2.9	0.326	0.43
Climbing ability boulder (IRCRA)	21.4 ± 3.6	18.1 ± 3.3	0.036	**0.94**
Experience (years)	12.1 ± 7.6	8.3 ± 4.1	0.188	**0.59**
Climbing-specific training (h/week)	6.7 ± 4.7	5.3 ± 1.8	0.445	0.35
Endurance training from total climbing time (%)	55 ± 28	64 ± 32	0.486	0.31
F_max_ (kg)	57.5 ± 11.2	38.0 ± 8.3	**<0.001**	**1.88**
CA mathematical model (°)	−2.5 ± 4.3	−2.6 ± 2.1	0.990	0.01
CA NIRS (°)	−2.7 ± 3.0	−2.3 ± 2.7	0.728	0.15
Peak angle (°)	−16.7 ± 5.3	−16.5 ± 4.5	0.913	0.05
W′ (°s)	3,491 ± 1,303	2,685 ± 1,455	0.168	**0.60**

*Statistically (p < 0.05) significant, and effect sizes greater than medium (d > 0.5) are in bold format.*

*CA, estimated critical angle; IRCRA, International Rock Climbing Association; F_max_, maximal finger flexor strength; NIRS, near infrared spectroscopy.*

### Procedures

All participants completed several exhaustive climbing tests during 5–7 laboratory visits separated by 2–5 days. During visit one, climbers undertook a maximal finger strength test and a familiarization session on the motorized climbing ergometer (treadwall) at several speeds and angles on a predetermined route. This route was also subsequently used for the exhaustive testing protocol. On visit two, climbers performed an incremental exhaustive exercise test, which progressed from a positive angle (+6°), through vertical (0°) to negative (overhanging) angle, the angle at which failure occurred was termed the “peak-angle.” Climbers were fitted with a NIRS device on their forearms to assess muscle oxygen dynamics. During the next 3–5 visits, one of the preset angles was climbed at a constant speed until failure so that TTE occurred between 2 and 15 min (Vanhatalo et al., 2011; Jones et al., 2019). Furthermore, the TTE range between the steepest and the least steep angle was aimed to be as broad as possible (8–12 min) (Jones et al., 2019).

Moreover, to validate the CA determination from the mathematical model, nine participants completed two additional laboratory visits to climb the same route 2° above and below CA in randomly assigned order.

### Finger Strength

Maximal finger flexor strength was assessed on a climbing-specific dynamometer using methods previously shown to be reliable (Baláš et al., 2018; Michailov et al., 2018). Climbers were asked to progressively transfer their maximum weight (“hang”) on a wooden rung (23 mm deep) for 5 s with their dominant hand. Maximal strength was determined as the highest (peak) value from two trials.

### Climbing Tests

Climbing tests were conducted on a motorized treadwall (ClimbStation generation 1, Forssa, Finland). The route was technically simple with positively oriented and slightly crimped holds (2–3 cm size depth which enabled both the open and half-crimp grip positions) and was graded 8 on IRCRA grading scale at vertical angle (0°) by a professional routesetter. During all ascents, a speed of 9 m⋅min^–1^ was applied to minimize the opportunity for static resting positions during the climbs (Baláš et al., 2021). The incremental test started at +6° (positive angle), and after each minute, the belt was stopped for 10 s to allow climbers to dry their hands with chalk, following which the angle was decreased by −3° to become progressively vertical (0°) and then negative (overhanging), therefore requiring progressively greater finger flexor and upper-body strength involvement. Climbers were not allowed to touch the ground during rest periods. The exhaustive tests at given angles were completed at the same speed and the angle of each remained constant during the whole ascent. Participants were verbally encouraged to climb for as long as possible. Each test ended when a climber reached volitional exhaustion and stepped onto the safety mattress.

### Muscle Oxygenation Breakpoint

**FIGURE 1:**
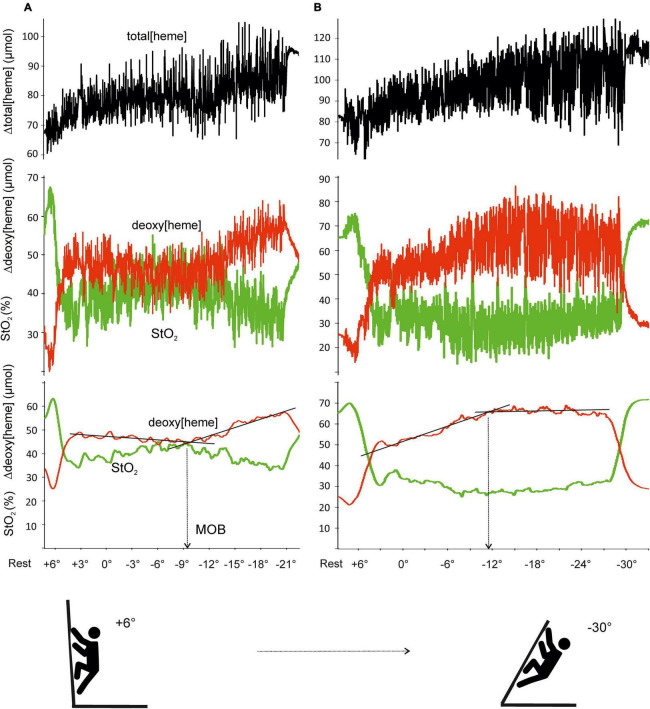
Example of raw and averaged (10-s interval) signal for deoxy[heme], total[heme], and muscle tissue oxygen saturation (StO_2_) during exhaustive climbing test with progressive increases in angle. MOB was detected as an inflection point of deoxy[heme] with progressive wall angle. Two typical responses represent sudden increase **(A)** and onset of a plateau **(B)** in Δdeoxy[heme].

During all ascents, a NIRS device (Portamon, Artinis Medical System, BV, Netherlands) was placed over the belly of the flexor digitorum profundus (FDP) (Fryer et al., 2018) and covered by a black forearm garment to shield the optodes from ambient light. Deoxy[heme], muscle tissue oxygen saturation (StO_2_), and total[heme] were used to assess muscle oxygen dynamics and perfusion. Due to the irregular intermittent nature of finger flexor contractions during climbing, deoxy[heme] and StO_2_ were averaged over 10-s periods. Raw and corrected NIRS signals are depicted in Figure 1. The MOB was determined visually from deoxy[heme] inflection points by three independent evaluators (Figure 1). The changes in slope signify that Δ deoxy[heme] had begun to change faster or slower with increased wall angle. If there was not an agreement on a determined CA, the following procedures were used: (1) if two evaluators were in agreement and one not, then the CA from two evaluators was used; (2) if all three reviewers were differing, then the mean score was used as the CA.

### Perceived Exertion

Rate of perceived exertion (RPE) during the ascents 2° above and under CA was used to assess subjective perception of exertion intensity. Perceived exertion was assessed on a scale from 6 to 20 as suggested by Borg (1982). Immediately after the test, climbers were shown a table with numbers and corresponding verbal description of the exertion and indicated their exertion rating to the researcher.

### Statistical Analysis

**FIGURE 2:**
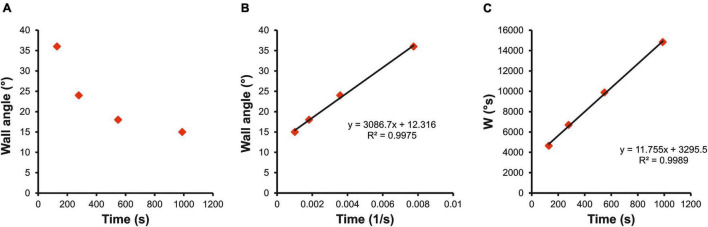
Example of hyperbolic relationship between wall angle and TTE **(A)** and the calculation from linear models using **(B)** wall angle (°) against time to failure (1/s); and **(C)** work limit W(°s) against time to failure (s). Coefficient of determination (*R*^2^) indicates the fit of the linear model.

Performance and NIRS characteristics were described using mean ± standard deviation (SD). Possible differences between men and women were evaluated using independent *t*-tests and Cohen’s *d*. To calculate CA, a similar approach for CF was applied (Giles et al., 2019) and the equation with best fit was used for determination of CA:


$$1.\;A=W^{\prime }\times \frac{1}{TTE}+CA,$$

$$2.\;W=\text{TTE}\times \text{CA}+{\text{W}}^{\prime },$$

where “A” is the angle of the ascent (°), “CA” is the critical angle (°), “TTE” is the time to exhaustion (s), “W′” is the capacity to climb over CA (°s) and represents the finite time a climber can sustain the ascent at steeper angles than CA, while “W” (°s) can be approximated as “total work” completed by a climber during the incremental exhaustive test. This first equation model plots angle of the ascent against l/TTE (Figure 2B); CA is given by the *y*-intercept and W′ by the slope of the regression line. The second equation model plots W against TTE (Figure 2C); CA is given by the slope of the regression line and W′ by the intercept. Both models were applied to all participants, and a model with higher fit was used to estimate individual CA.

To determine validity of CA from the mathematical model, nine climbers were asked to climb 2° above and under CA. The limit of 2° was calculated as 95% confidence interval (95% CI) from standard error of CA estimate (SE = 1.1), therefore 1.96 SE (95% CI = ±2.2°).

Subsequently, the agreement between the CA determination from mathematical model and NIRS was evaluated using Bland–Altman plot and intraclass correlation (ICC). The ICC was calculated as follows:


$$ICC=\frac{MSB-MSW}{MSB+\left(k-1\right)MSW},$$

where MSB and MSW correspond to mean squares between and within subjects from a repeated measure ANOVA, respectively, and k is the number of trials (2 in this case). This equation encompasses both the variability due to systematic changes between trials and error variability. ICC was expressed with 95% CI.

The association among climbing ability, TTE, CA, and W′ were evaluated using Pearson’s correlation coefficients or linear regression coefficient of determination. Statistical significance was set to *p* < 0.05.

## Results

When TTE was plotted against wall angle, the typical hyperbolic function as for power–duration relationship was found (Figure 2A). The linear transformation (wall angle against 1/TTE, Figure 2B) showed high model fit (*R*^2^ = 0.99; 95% CI 0.96–1.00) and low standard error of CA estimate (SE = 1.10°; 95% CI 0.83°–1.35°). The second linear model (W against TTE, Figure 2C) provided less fit (*R*^2^ = 0.72; 95% CI 0.61–0.83), and a low standard error of CA estimate was found (SE = 0.99°; 95% CI 0.72°–1.25°).

Time to exhaustion at the steepest angle was 118 ± 52 s (wall angle range from −25° to −45°), and the least steep angle was 808 ± 192 s (wall angle range from 0° to −18°). The mean estimated CA (−2.5° ± 3.8°) was significantly associated with climbing ability in lead climbing but not in bouldering (*R* = −0.406 and −0.282, respectively); however, W′ (3251°s ± 1373°s) was related to both lead climbing and bouldering ability (*R* = 0.580 and 0.695, respectively).

The mean peak angle during the incremental test was −17° ± 5° and was moderately related to both lead climbing and bouldering ability (*R* = −0.661 and −0.587, respectively). Training and performance characteristics for both men and women are depicted in Table 1.

**FIGURE 3:**
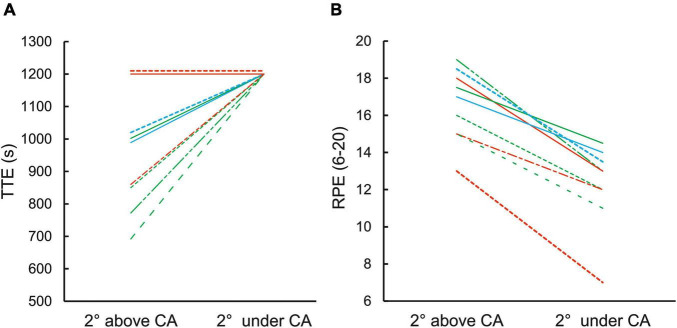
**(A)** TTE during climbing 2° above and under CA. The time of 20 min (1,200 s) was set as maximal irrespective exhaustion occurred or not. Immediately after the climb, RPE was assessed in both conditions **(B)**. Color of individual lines represents climbers with different level of W′: red (W′ > 3,500°s), blue (W′ > 2,500–3,500°s), green (W′ < 2,500°s).

All nine climbers performing the ascent 2° under CA were able to sustain the task for the maximum test duration of 20 min with perceived exertion (RPE = 12.1 ± 1.9). However, climbing 2° above CA led to task failure (TTE = 954 ± 177 s; RPE = 16.4 ± 1.9) (Figure 3). Only 2 climbers were able to sustain the task for 20 min which was in agreement with their exceptionally high W′ (W′ > 3,500°s) as their TTE was predicted to last more than 30 min (Figure 3).

The MOB was detectable in all 27 climbers during the incremental exhaustive test; 18 showed inflection points as a faster increase in Δdeoxy[heme], whereas 9 climbers as an onset of a plateau (Figure 1).

**FIGURE 4:**
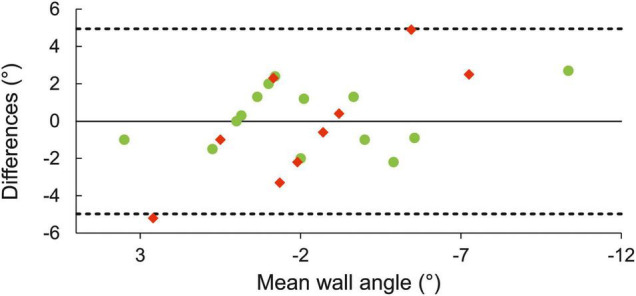
Limits of agreement plot of estimated CA from MOB and CA from mathematical model. The solid horizontal line represents differences between the two estimates of CA, the dashed lines upper and lower 95% limits of agreement. Green and red circles designate participants with MOB determined as a sudden increase and onset of a plateau in Δdeoxy[heme], respectively.

Good agreement was found between angle at MOB and CA (ICC = 0.80, 95% CI 0.61–0.90, SEM = 1.5°). Limits of agreement plot showed no meaningful differences between the two methods, and nearly all estimates were within ± 3° (Figure 4). The estimate of MOB as an onset of deoxy[heme] plateau provided larger variability than the inflection point of faster Δdeoxy[heme] increase (Figure 4).

## Discussion

The main findings of this study were that (1) multiple tests to exhaustion with differing climbing angles allow for the estimation of CA at which a maximal metabolic steady state occurs when climbing; (2) MOB representing a metabolic transition state in the finger flexors is in good agreement with CA.

Manipulating wall angle has previously been shown to be a simple quantitative tool for changing intensity in climbing (Watts and Drobish, 1998). Noé et al. (2001) reported that an increase of 10° (−10° from vertical) induced ∼47% increase in mean vertical force on handholds and, therefore, more intense finger flexor contractions. Furthermore, an increase of wall angle by 15° from vertical elevated heart rate by an average of ∼24 beats per min, oxygen uptake by ∼9 mL⋅min^–1^⋅kg^–1^ and lowered muscle oxygen saturation of the FDP by 7% (Baláš et al., 2021). In this study, TTE decreased with steeper wall angle and the association between TTE and wall angle followed a hyperbolic function (Figure 2) same to the power or speed and duration relationship (Jones et al., 2019). The existence of a CA as a metabolic transitional zone between steady- and nonsteady-state conditions provides further support for using climbing angle to adjust intensity during climbing training. However, it should be noted that the same wall angle may induce different forces on handholds even among climbers who have similar characteristics such as body mass and finger strength endurance. In fact, the whole-body model assessment may encompass not only the metabolic capacity of the forearm flexors but also other factors such as movement economy. For instance, movement economy in more advanced climbers has been shown to reduce vertical forces on handholds (Baláš et al., 2014), which would lead to a steeper CA in more “technical” than “stiff” climbers despite their similar metabolic predispositions. Consequently, comparisons among climbers of estimated CA account not only for the level of aerobic capacity, but also for other factors such as movement economy. This is in contrast to the isolated forearm model CF determination (Giles et al., 2019, 2020), where only metabolic factors in specific hanging conditions are assessed. However, the primary aim of the CA determination is not the between-subject comparisons but individual threshold intensity in ecological valid setting. Although movement economy may differ among climbers, the CA is set for each climber individually as it is expected that each climber has similar movement economy across all trials on the same route. Therefore, technically easy routes should be preferred for individual training prescription to ensure that the shift in CA is due to metabolic adaptations and not a learning effect. Moreover, it should be noted that values of estimated CA will be only valid for a predefined route, as different hold sizes, more complex moves, or different climbing speeds may induce different physiological responses. Using motorized treadwalls appear especially appropriate for the standardization of training, where the primary aim is to influence metabolic adaptations of finger flexors during whole-body climbing movement which may differ from isolated training on hangboards or campus boards (Medernach et al., 2015; Levernier and Laffaye, 2019; Giles et al., 2020).

Repeated exhaustive ascents over several days are needed to determine CA and this places high-training loads on individuals. Therefore, using MOB during one exhaustive incremental test may be more advantageous for trainers and climbers to set a climbing-specific maximal metabolic steady state. The MOB during an exhaustive climbing incremental protocol has been described previously (Baláš et al., 2021); however, the authors could not associate the inflection point of Δdeoxy[heme] to any intensity threshold as no relationship to any ventilatory or cardiac responses was found. In this study, the MOB comparison was made with the CP concept as the local forearm muscle fatigue rather than respiratory exhaustion is the main determinant of failure during climbing (Watts, 2004). We found good agreement between MOB and CA (SEM = 1.5%), particularly considering 3° was the smallest change used during the incremental test. However, there were 2 of the 27 climbers who demonstrated differences of ∼5°. The explanation may be linked to several mechanisms such as reliability error, error of CA determination or simply that the MOB cannot precisely reflect metabolic steady-state intensity. Therefore, repeated testing or climbing slightly below the CA for extended periods of time appears useful to confirm the correct determination of CA.

Deoxy[heme] has been recommended for MOB determination as it is less affected by changes in perfusion under NIRS probe (Grassi et al., 2003; Wang et al., 2006). Two patterns in Δdeoxy[heme] dynamics have been revealed to represent MOB in the current results in line with the literature (Wang et al., 2006). First, the onset of a plateau in deoxy[heme] may reflect microvascular O_2_ extraction reaching a ceiling (Keir et al., 2015; Boone et al., 2016a) or simply that short periods of finger flexor reperfusion during hand release from the hold are not sufficient to provide sufficient blood flow at higher intensities and then O_2_ delivery into the muscle. Only 9 climbers in this study showed plateau of deoxy[heme], and the other 18 climbers demonstrated faster increase in Δdeoxy[heme] at MOB. According to our data (Figure 4), both forms of inflection reflected similar intensities around the CP. This discrepancy in the oxygen dynamics between climbers may be due to many interrelated factors such as relative deepness of muscle analyzed under optodes (climbers had various forearm circumference), the muscles involved in the contraction (muscle fiber architecture), muscle fiber types assessed, and/or blood perfusion during the test (Chin et al., 2011; Murias et al., 2013; Okushima et al., 2015). For instance, it has been demonstrated that the deeper layers of rectus femoris have the potential to maintain a higher O_2_ delivery to O_2_ utilization ratio compared with superficial layers during incremental cycling (Okushima et al., 2015). The less activated rectus femoris provides right-shifted dynamics of deoxy[heme] with respect to a more involved vastus lateralis and vastus medialis during ramp exercise (Chin et al., 2011). It is likely that other finger flexors such as the superficial flexors may have been largely involved during intermittent contractions and mitigated the activity of deeper flexors.

In this study, good agreement between MOB and CA as the maximum steady-state intensity was found. Moreover, all climbers exercising 2° under CA were able to sustain 20 min of climbing rating the intensity from light to somewhat hard on Borg scale of perceived exertion, while they were exhausted 2° above CA after ∼ 16 ± 3 min rating the intensity from hard to extremely hard. This supports our hypothesis that MOB during isometric contractions reflects metabolic changes in the muscle from steady- to nonsteady-state conditions, rather than other intensity boundaries. However, it should be acknowledged that NIRS-derived thresholds may be only mechanistically linked to CP threshold as discussed recently (Boone et al., 2016b; Broxterman et al., 2018; Poole et al., 2021).

There was a significant but practically weak relationship between CA and climbing ability (*R*^2^ = 0.16) which is in contrast to moderately strong association (*R*^2^ = 0.66) from similarly determined MOB in our previous study (Baláš et al., 2021). The discrepancy may be due to selection of climbers who were mixed in sex, and discipline preference. This is supported by generally lower relationship between peak angle and climbing ability in this study when compared to previous research (España-Romero et al., 2009; Baláš et al., 2021). However, the significant relationship between CA and lead climbing ability supports the importance of oxidative capacity for achieving a high level of performance in lead climbing. On the other hand, a weak nonsignificant association (*R*^2^ = 0.08) with bouldering ability shows that other factors are decisive for the performance of powerful whole-body movements in bouldering. For instance, the ability to climb at intensities (angles) above CA (W′) has been shown to be a more important metabolic determinant for bouldering (*R*^2^ = 0.48) than for lead climbing (*R*^2^ = 0.33). With respect to this study, it has to be highlighted that climbing performance depends on many other technical and tactical factors and the association between CA and climbing ability will always be lower than typical endurance sports such as running or cycling (Poole et al., 2021). Nevertheless, endurance of the finger flexors is a key sport-specific determinant of performance and lead climbers demonstrate specific adaptations (Ferguson and Brown, 1997; Thompson et al., 2014). It has been suggested that using CP and W′ may be extremely valuable in constructing individually optimized interval training programmes in a range of athletes and sport disciplines (Vanhatalo et al., 2011); therefore, coaches may use CA as the threshold intensity to train forearm muscles endurance under sport-specific conditions.

Limitations of the study include the assumptions associated with the use of continuous-wave NIRS measurement during exercise such as adipose tissue thickness, subcutaneous blood flow, or the use of physiological calibration (Barstow, 2019). However, adipose tissue under the optodes should not have affected the results as skinfold thickness in climbers’ forearms has been found to be very low (Baláš et al., 2018; Fryer et al., 2018). In addition, the use of spatial resolved spectroscopy, as used in this study, appears to be unaffected by heating-induced changes in cutaneous circulation (Barstow, 2019), and as such there was no need for the physiological calibration of the NIRS output as changes in TSI and deoxy[heme], rather than absolute values, were evaluated (Barstow, 2019). It should also be noted that the findings of this study are based on a technically simple climbing route at one speed with handholds of a relatively similar size, which may differ from technical rock-climbing ascents.

## Conclusion

Our data show that multiple tests to exhaustion with differing climbing angles allow for the estimation of CA. Climbers, coaches, and researchers may use a predefined route or circuit at three to five angles to estimate CA as a parallel of metabolic transition from steady to nonsteady states (heavy to severe exercise intensity domains). Climbing 2° below CA is tolerable for extended periods of time and perceived as light to somewhat hard, while climbing 2° above CA leads to finite time to failure. Moreover, an exhaustive climbing test with progressive increases in angle using the MOB appears to provide a valid estimation of CA.

## Data Availability Statement

The raw data supporting the conclusion of this article will be made available by the authors, without undue reservation.

## Ethics Statement

The studies involving human participants were reviewed and approved by Ethics Committee of Charles University, Faculty of Physical Education and Sport. The patients/participants provided their written informed consent to participate in this study.

## Author Contributions

JB developed the theoretical framework, conceived the study, collected the data, analyzed the data, and wrote the article. JG developed the theoretical framework, conceived the study, and analyzed the data. SF and DG provided critical feedback on drafts and edited the final manuscript for submission. All authors contributed to the article and approved the submitted version.

## Conflict of Interest

DG is employed by Lattice Training Ltd., who provides climbing coaching and assessment services. JB is currently affiliated with Climbro, a private company who provides hangboards with integrated force sensors and mobile application for climbing specific training. The remaining authors declare that the research was conducted in the absence of any commercial or financial relationships that could be construed as a potential conflict of interest.

## Publisher’s Note

All claims expressed in this article are solely those of the authors and do not necessarily represent those of their affiliated organizations, or those of the publisher, the editors and the reviewers. Any product that may be evaluated in this article, or claim that may be made by its manufacturer, is not guaranteed or endorsed by the publisher.
